# Supplementary figures and images for: An image-based assay to quantify changes in proliferation and viability upon drug treatment in 3D microenvironments
Source: BMC Cancer. 2019 May 28;19:502. doi: 10.1186/s12885-019-5694-1 (PMC6537405; doi:10.1186/s12885-019-5694-1)

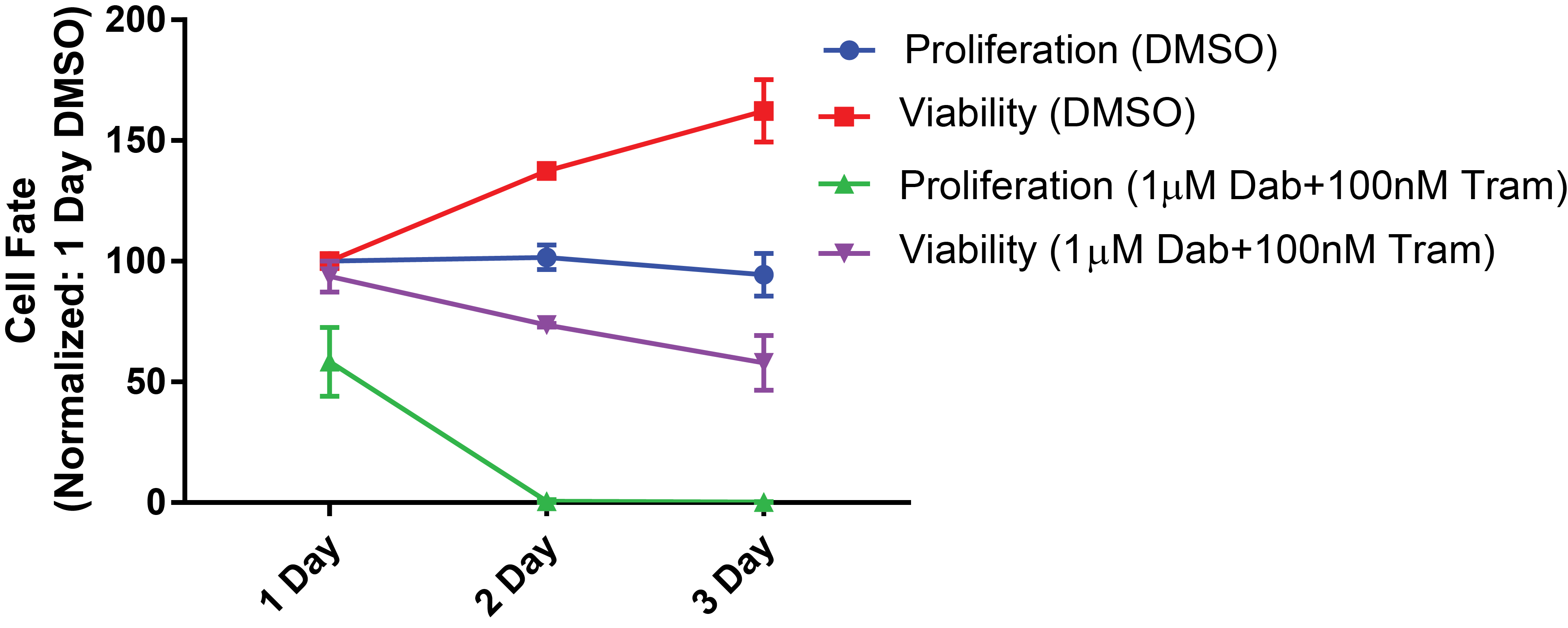

Supplement: Supplementary file 1 — Figure S1. Cell profiler pipeline for image analysis Image analysis using cell profiler involved thresholding, followed by segmentation to identify pixels to identify the total image area occupied for each channel. The intersection between EtHd and Apopxin was used to exclude pixels positive for both markers from being counted twice. (PNG 138 kb) [file 12885_2019_5694_MOESM1_ESM.png]

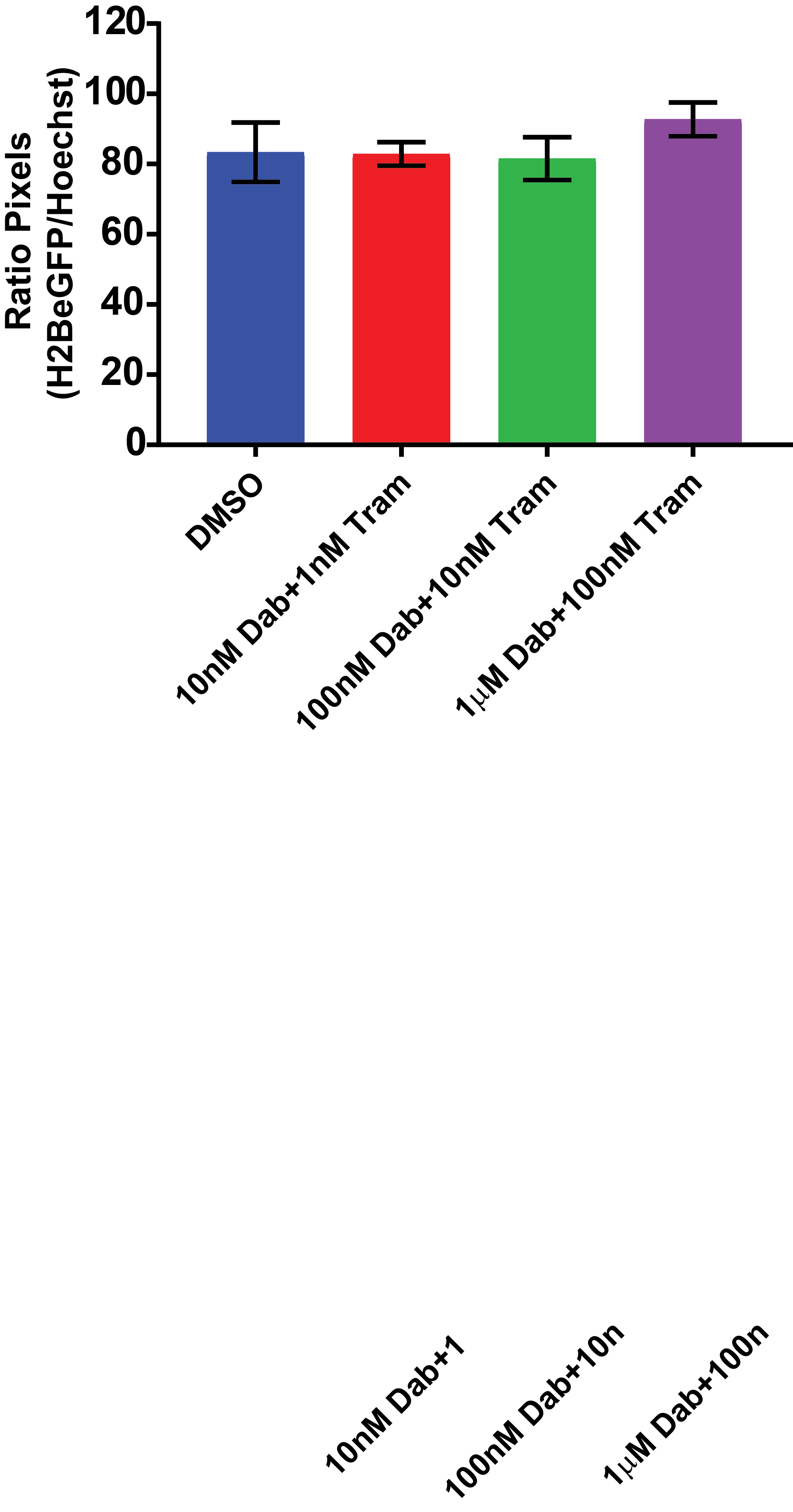

Supplement: Supplementary file 2 — Figure S2. Comparison of results analyzed using Cell Profiler to analysis by python image analysis. Data from experiments using A375 (A) M481 (B) and M498 (C) cells treated with RMIC were analysis using both image analysis pipelines. Data shown are three independent experimental repeats with results presented as average ± SEM. (* p value < 0.05). (PNG 120 kb) [file 12885_2019_5694_MOESM2_ESM.png]

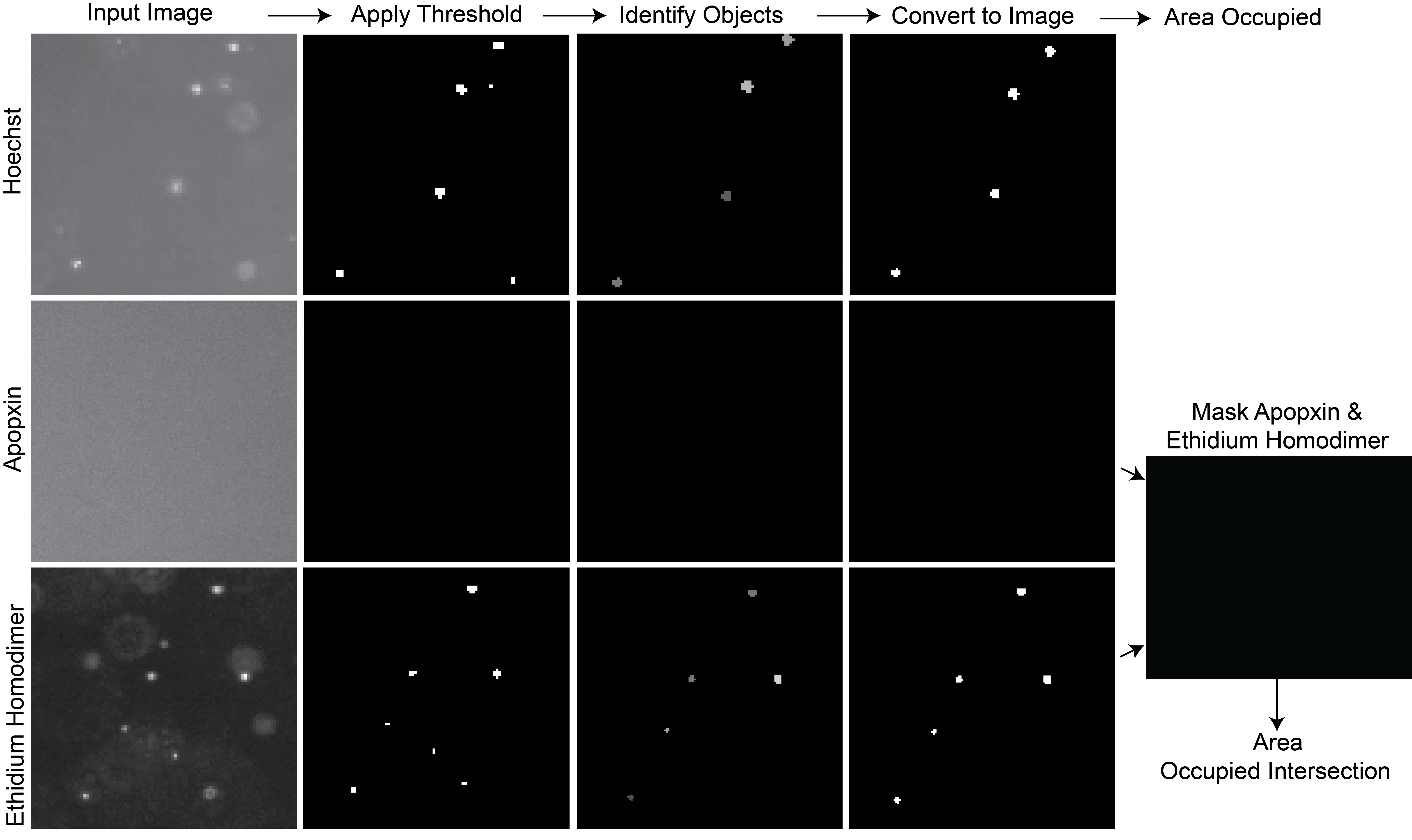

Supplement: Supplementary file 3 — Figure S3. Time course of the effect of RMIC on proliferation. A375 cells treated with RMIC at a concentration of 1 μM Dab + 100 nM Tram in 3D were incubated for 1, 2 and 3 days. (PNG 355 kb) [file 12885_2019_5694_MOESM3_ESM.png]

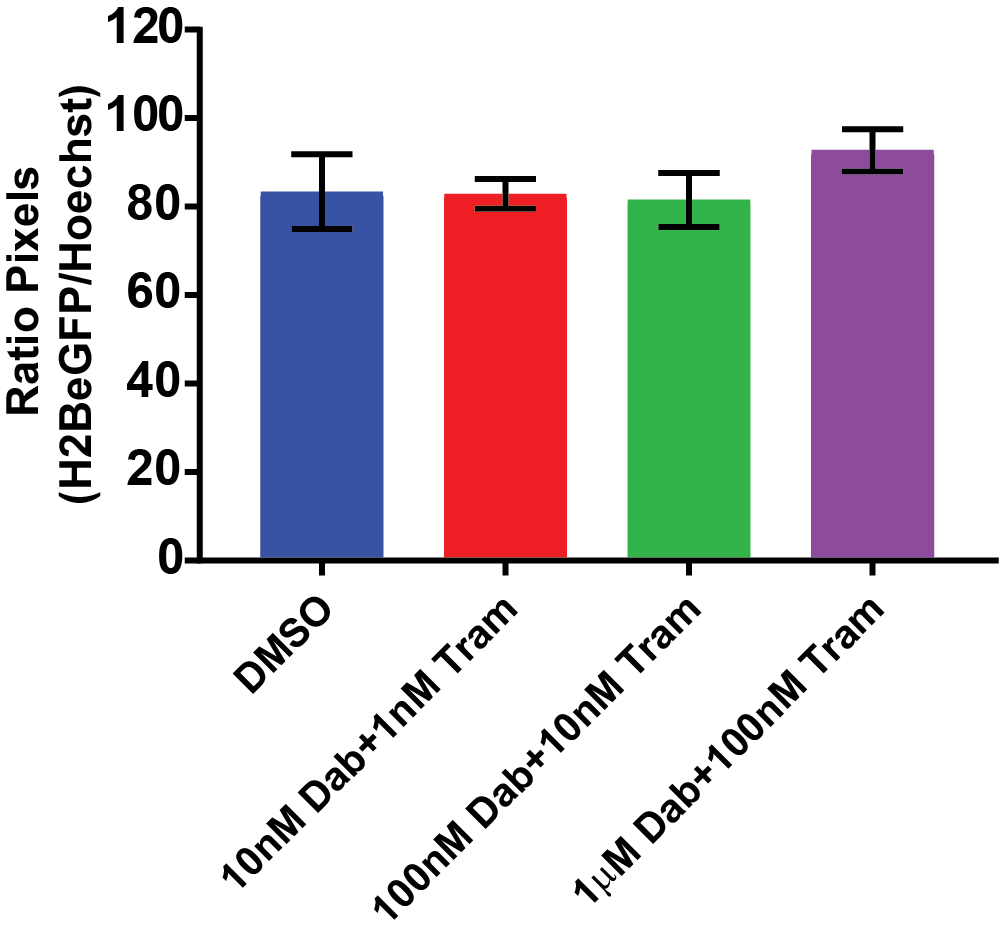

Supplement: Supplementary file 4 — Figure S4. Validation of the image based assay for identifying fibroblasts in 3D culture. HFF1-H2BeGFP cells in 3D collagen were treated with RMIC as a function of concentration and pixels positive for GFP were compared to pixels positive for Hoechst to determine the overlap between GFP cells and Hoechst positive cells. (PNG 33 kb) [file 12885_2019_5694_MOESM4_ESM.png]
